# A Markerless Approach for Full-Body Biomechanics of Horses

**DOI:** 10.3390/ani15152281

**Published:** 2025-08-05

**Authors:** Sarah K. Shaffer, Omar Medjaouri, Brian Swenson, Travis Eliason, Daniel P. Nicolella

**Affiliations:** Southwest Research Institute, 6220 Culebra Rd., San Antonio, TX 78238, USA

**Keywords:** equine, motion capture, machine learning, kinematics

## Abstract

The ability to quantify equine kinematics is essential for clinical evaluation, research, and performance feedback. However, current methods are challenging to implement. This study presents a motion capture methodology for horses, where three-dimensional, full-body kinematics are calculated without instrumentation on the animal, offering a more scalable and labor-efficient approach when compared with traditional techniques. Kinematic trajectories are calculated from multi-camera video data. First, a neural network identifies skeletal landmarks (markers) in each camera view and the 3D location of each marker is triangulated. An equine biomechanics model is scaled to match the subject’s shape, using segment lengths defined by markers. Finally, inverse kinematics (IK) produces full kinematic trajectories. We test this methodology on a horse at three gaits. Multiple neural networks (NNs), trained on different equine datasets, were evaluated. All networks predicted over 78% of the markers within 25% of the length of the radius bone on test data. Root-mean-square-error (RMSE) between joint angles predicted via IK using ground truth marker-based motion capture data and network-predicted data was less than 10 degrees for 25 to 32 of 35 degrees of freedom, depending on the gait and data used for network training. NNs trained over a larger variety of data improved joint angle RMSE and curve similarity. Marker prediction error, the average distance between ground truth and predicted marker locations, and IK marker error, the distance between experimental and model markers, were used to assess network, scaling, and registration errors. The results demonstrate the potential of markerless motion capture for full-body equine kinematic analysis.

## Simple Summary

Equine gait data can be used to identify lameness and provide sports feedback. However, most tools that measure how a horse moves are time-consuming to use and require specialized expertise and equipment. This study introduces a method to track full-body, three-dimensional equine motion using videos collected by multiple cameras. Unlike traditional methods, no instrumentation needs to be placed on the horse. A neural network is used to identify the location of anatomic landmarks in the videos (e.g., the horse’s fetlock) and these predictions are tied to a musculoskeletal model. This process allows joint angles, stride information, and other motion data to be quickly computed. Our results show that most joint angles calculated by this method are within 10 degrees of those determined using data collected by a traditional system. We were able to process 1 min of video data, collected by 10 cameras, and produce results in less than 10 min. When these data were originally collected, it took 2–3 h to instrument the horse and additional time to process. With further improvements to increase accuracy, this methodology will make it easier and more affordable to collect full body movement data from horses.

## 1. Introduction

Quantitative gait analysis (QGA) is gaining popularity for use in equine health and athletic performance applications. For example, identification of lameness is a common use case for QGA as lameness can be challenging for veterinarians to diagnose and monitor [1,2,3,4]. Traditional lameness evaluations include a visual examination and subjective grading on a standardized scale [2,5]. These evaluations can be unreliable, as subtle gait abnormalities are difficult for humans to see and grading scales do not consider the full spectrum of lameness presentations [2,6,7,8]. Finally, these examinations may be influenced by knowledge of previous treatments [7,9]. QGA mitigates these issues by providing repeatable quantification of gait, detecting subtle changes in gait, and reducing expectation bias [2,6,7,8,9,10,11,12,13,14,15,16,17]. For these same reasons, QGA can be used to give feedback on athletic performance, quantify horse–rider interactions, and assist with fit-to-compete evaluations [18,19]. In the future, QGA could also be used to help train, evaluate, and support judges in equine sports.

Quantitative gait analysis techniques measure motion (kinematic methods) and/or forces (kinetic methods). Kinetic methods are generally limited to research settings [2,7]. Kinematic methods are more accessible but have practical issues that hamper widespread adoption [2,7,15,20]. The gold standard used to measure equine kinematics is marker-based motion capture (MBMC) [7]. MBMC requires markers placed over skeletal landmarks; marker motion is tracked by specialized cameras and converted to biomechanical data. MBMC traditionally requires a laboratory space and data collection is time consuming [2,20]. Inertial measurement unit (IMU) systems are also used to detect movement asymmetries, motion of body segments, and stride parameters, but are rarely used to capture full-body kinematics [2,6,10,11,12,16,17,18]. Other issues that hamper the widespread use of MBMC and IMU systems are that data are sensitive to device placement, outputs are limited to labeled body segments, and the devices may interfere with movement or equipment, affect animal behavior, and become dislodged during motion [18,21,22]. A recent survey indicates that veterinarians wish for more measured parameters, increased functionality, and easier-to-use QGA technologies [15].

Markerless motion capture (MMC) is an easy-to-use alternative available for humans. MMC allows kinematics to be determined from videos, without markers or sensors on the subject. Machine learning techniques predict the location of markers on an individual or fit a shape model to an individual in images [20]; these predictions can be coupled with a biomechanical model to constrain motion to realistic trajectories, simulate ground-surface interactions, and predict skeletal forces.

Existing equine MMC systems generally do not supply full body kinematics; most use 2D representations to determine univariate measures of gait (e.g., stride length, movement asymmetries) [16,17,23,24,25,26,27]. However, recent work has shown that accurate equine 3D shape and pose representations can be built [28,29,30,31,32]; these parametric models encode three-dimensional (3D) motion data and show promise in predicting lameness [28]. However, to our knowledge, a direct comparison of the full-body kinematic trajectories predicted by these MMC methods to MBMC data has not been performed.

An equine MMC system capable of predicting full-body kinematics would reduce the burden of traditional analysis, while offering more functionality and derived parameters compared with existing systems. Full-body kinematic data can provide metrics already in use for lameness identification (e.g., landmark asymmetry), performance feedback, and clinical research (e.g., joint angles). Easy scalability of MMC would also allow data to be explored with methodology that has not been easily accessible to the veterinary community. For example, full-body kinematics from multiple movements, measured prior to training, are more useful in predicting which military recruits will sustain an injury during an intense training program than traditional univariate measures [33]. These methodologies (and others) could be leveraged by the veterinary community with easier access to full-body kinematic data.

Our goal was to demonstrate that 3D MMC is possible in horses. We adapted a human MMC software [34] by leveraging a dense equine MBMC dataset [29], training a neural network to predict markers, and linking marker predictions to an equine musculoskeletal model. We then conducted a brief validation of the developed MMC pipeline using MBMC data as ground truth.

## 2. Materials and Methods

**Figure 1:**
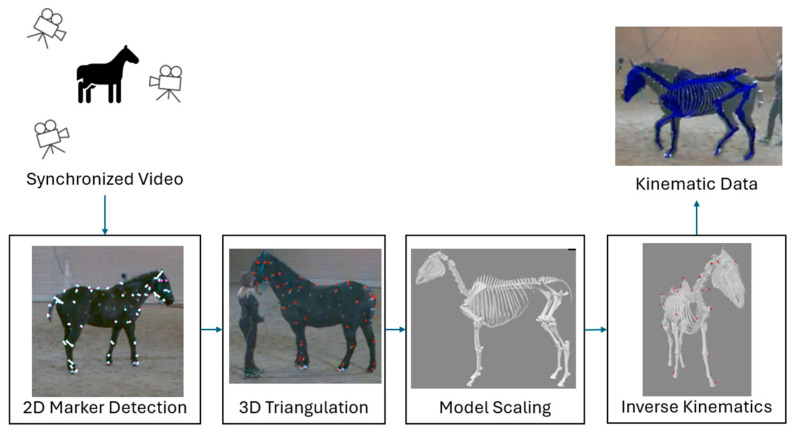
Data processing pipeline; example data from [29].

A multi-step pipeline, based on the Engine for Automated Biomechanical Analysis (ENABLE), was used to determine 3D kinematics from multi-camera video data (Figure 1). ENABLE is validated for human use and computes biomechanical data in real-world environments [34,35]. In step 1, the 2D location of skeletal landmarks (markers) are identified in each camera view by a convolutional neural network (CNN). In step 2, triangulation is performed to reconstruct the 3D location of each marker using the 2D predictions and camera calibration information. In step 3, a musculoskeletal model is regionally scaled using predicted landmarks. In step 4, inverse kinematics is performed on the musculoskeletal model to produce kinematic trajectories.

### 2.1. Equine Datasets

**Figure 2:**
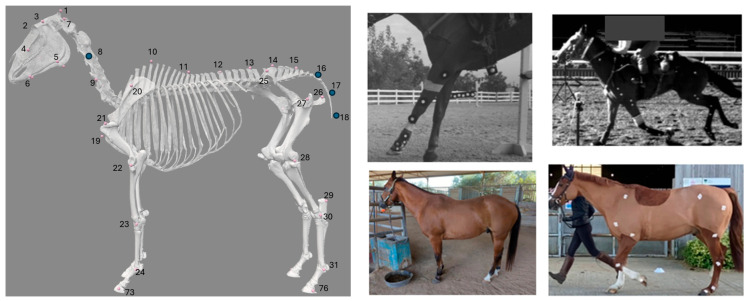
(**Left**) Virtual markers (pink or blue circles) on the musculoskeletal model; markers 3–9, 20–31, 73, and 76 are duplicated on the left and right side. The following anatomic locations have a marker: poll (1), forehead (2), temple (3), cheek (4), jaw (5), chin (6), three locations on the side of the neck (7–9), withers (10), vertebral markers (11–18), center of the chest (19), shoulder blade (20), shoulder joint (21), elbow joint (22), carpal joint (23), forelimb fetlock joint (24), and forelimb hoof (73), tuber coxae (25), ischiatic tuberosity (26), hip joint (27), stifle joint (28), point of hock (29), hock joint (30), hindlimb fetlock joint (31), and hindlimb hoof (76). Blue markers were not used during inverse kinematics. (**Right**) Example images in the SwRI_Horse dataset [36,37,38]. These datasets primarily consist of marker-based motion capture (MBMC) data.

Three datasets were used: SwRI_Horse (developed for this study), PFERD_base (data from The Poses for Equine Research Dataset (PFERD) [29]), and PFERD-SwRI_Horse (a combination of the two). Each dataset consists of images labeled with the location of skeletal markers (Figure 2) and a rectangular region of interest (ROI) around the horse. Fifty-four skeletal landmarks were labeled with a marker (Figure 2). This marker set is a subset of the set defined in PFERD and uses consistent numbering and naming conventions [29].

SwRI_Horse contains labeled data from more than 500 horses in a variety of environments (Figure 2). It includes MBMC data from university labs (~69,000 images; 18 horses), open-source data (1498 images; >500 horses), and hand-labeled data collected at a local barn (362 images; 7 horses) [26,36,37,38,39,40]. The MBMC data [36,37,38] consist of images (i.e., individual video frames) and the location of each skin-fixed marker identified by the original MBMC system. Additional markers on the limbs were added to our marker-set to incorporate the Rohlf and Symons marker sets. Open-source data were primarily hand-labeled as described in [26,39,40]; only horse data were used when other species were present in these datasets and only keypoints matching markers in our marker set were used. Used images were screened to ensure labeling was consistent with our marker set. Due to disparate sources and camera views, not all images are completely labeled with the full marker set.

The SwRI_Horse dataset was not used in isolation during this study because most images were side views (Figure 2) and a CNN trained on this data could not perform well on the other views required for 3D triangulation. For this same reason, horses in this dataset could not be used to validate the full pipeline (e.g., predict 3D kinematics). So, it was combined with PFERD_base to create PFERD-SwRI_Horse.

The Poses for Equine Research Dataset (PFERD) includes detailed 3D MBMC data projected into 2D images frames from 10 cameras [29]. Data were collected on 5 horses (PFERD Horse ID 1–5) of different breeds performing various movements in an indoor arena [29].

### 2.2. Skeletal Marker Predictions

The HR-Net architecture [41] was selected for our neural network that predicts the 2D location of skeletal markers; this architecture consists of multiple parallel branches at different feature resolutions, thus preserving fine-grain visual features without sacrificing performance. The network produces 2D pose predictions in the form of multi-channel heatmaps with each channel containing a 2D distribution of the likelihood a specific keypoint is present at a given pixel coordinate [42]. Predicted 2D keypoint locations are then obtained by locating the maximum activation within each channel.

Predictions of the 3D locations of the skeletal markers are performed as follows using trained networks: A pretrained version of the standard MaskRCNN [43] object detection network provides the initial (first frame) horse region of interest (ROI). Then, the network predicts the 2D location of each skeletal marker. Once this is completed for each camera view, a triangulation process determines the 3D locations of each marker. The triangulation procedure consists of a random sample consensus (RANSAC) algorithm [44] which takes pairs of 2D skeletal markers and computes 3D rays originating at their respective source camera using the intrinsic parameters (*K*, the intrinsic matrix and *d*, the distortion coefficients) and extrinsic parameters (*R*, the rotation matrix, and *t*, the translation vector) of each camera, obtained during camera calibration [45]. These ray pairs are used to obtain candidate 3D locations for each skeletal marker by locating the nearest point to both rays. The RANSAC algorithm calculates the number of inliers and outliers for each candidate 3D location and selects the candidates which minimize the number of outliers, thus reducing the impact of spurious 2D marker predictions. The resulting 3D marker locations are then reprojected into each camera view and used to help derive the horse ROI on the following frame. This process is repeated until the entire capture (all video frames) is processed.

An identical network architecture was trained on the PFERD_base and PFERD-SwRI_Horse datasets. The PFERD_base dataset was partitioned into a training split (PFERD ID 1, 2, 4), a validation split (PFERD ID 3), and a testing split (PFERD ID 5). The combined PFERD-SwRI_Horse model was trained with the same configuration with the SwRI_Horse dataset divided between training and validation splits. The testing split (PFERD ID 5) was used to assess 2D performance of each network and predicted kinematics from our full pipeline.

For all network training and evaluation, an NVIDIA L40S was used. The AdamW optimizer was used for network training due to its effectiveness in handling sparse gradients and improving convergence. For both datasets, the network was trained for 30 epochs with a batch size of 40 samples. An initial learning rate of 0.0001 was used and adjusted to ensure stable convergence. The loss metric employed during training was mean squared error, which measures the difference between the predicted heatmaps and the ground truth heatmaps, allowing the model to minimize the pixel-wise error between the predictions and ground truth. These ground truth heatmaps were generated using the horse poses in each dataset, ensuring that the network could learn to accurately predict the spatial distribution of key points. A standard set of data augmentation techniques was used, which included randomized rotation and scaling.

The 2D performance of our horse pose estimation network was evaluated using the percentage correct keypoints (markers) within a threshold distance (PCKh) [46] for each network. The right radius length, defined as the distance between the right carpal and elbow joint (markers 23 and 24; Figure 2), was used as our PCK threshold distance. This segment offered greater stability than the head segment, which is typically used in human studies, due to variability in how the head segment was projected into 2D. The right radius length was between 23–47 cm for horses in the PFERD set and has a more general range of 32–40 cm for riding horses [29,47]. We computed our PCKh metric at the 10% (PCKh0.1), 25% (PCKh0.25), 50% (PCKh0.5), and 100% (PCKh1.0) threshold levels. The reported PCKh indicates the percent of markers correctly predicted within the specified percentage of the threshold distance to the ground truth marker location.

### 2.3. Equine Biomechanics Model

**Figure 3:**
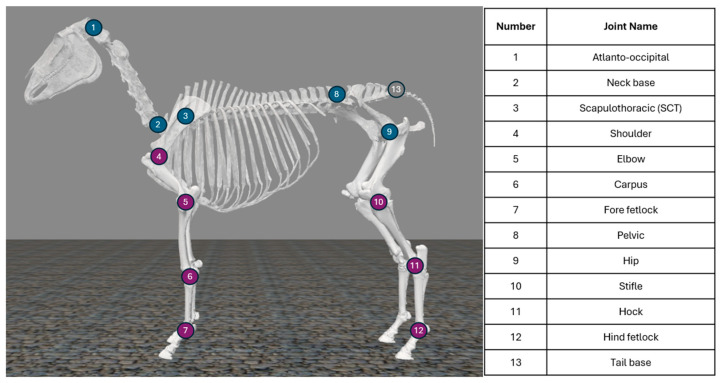
Equine musculoskeletal model; adapted from [48]. Joints have either 3 rotational degrees of freedom (blue), 1 rotational degree of freedom (purple), or are fused (grey). Limb joint angles are defined so that the long bones are aligned at a 0° flexion/extension angle, with extension as a positive angle and flexion as a negative angle. The default orientation (shown) has the SCT at +34°, shoulder at −73°, elbow at −40°, carpus at 0°, fore and hind fetlock at +30°, hip at −60°, stifle at −54°, and hock at −24°. The atlanto-occipital joint, neck base, and pelvic joints are shown at 0° rotation about their local axes.

An open-source equine biomechanical model developed in OpenSim was used as a base model [48]. Degrees of freedom (DOF) were added to allow movement outside the sagittal plane and articulation at the pelvis. In our updated model, the torso has 3 translational and 3 rotational DOFs relative to the global reference frame and there are 23 joints with a total of 35 rotational DOFs (Figure 3). Joint range of motion (ROM) was restricted to within physiological limits [38,49,50] or from more than 180-degree rotation, if a literature reference was not found. Musculature is included in this model [48] and could be leveraged in the future. Virtual markers corresponding to the defined marker set were fixed on the model (Figure 2).

### 2.4. Pipeline Validation

Data from the horse in the network testing split (PFERD ID 5 [29]) were used for a validation of the full MMC pipeline (Figure 1). As this horse was in the network testing split, trained networks had not seen images of this horse prior to pipeline validation. Data consisted of 14 strides at the walk, 24 strides at the trot, and 8 strides of canter on a small circle (PFERD ID 5, Trial 3; MBMC data was downsampled in [29] to match color video frame rate). The validation horse was missing the forehead and temple markers in its ground truth data [29], so, these markers were excluded during analysis.

Five metrics were computed to validate the full MMC pipeline: prediction error, IK marker error, joint angle root mean square error (RMSE), Pearson’s correlation coefficient, and interclass correlation coefficient. These metrics were calculated as follows. First, network predicted marker trajectories were filtered with a zero-phase 6th-order Butterworth low-pass filter with a cut-off frequency of 4Hz. Prediction error, the distance between the ground truth and filtered predicted marker locations, was determined for each marker at each timepoint (Equation (1)).

(1) $$\mathrm{Prediction}\;\mathrm{Error}\;=\;\sqrt{{\left(X_{GT}-X_{NN}\right)}^{2}+{\left(Y_{GT}-Y_{NN}\right)}^{2}+{\left(Z_{GT}-Z_{NN}\right)}^{2}}$$

where GT = MBMC data (ground truth) and NN = network predicted data

Next, the musculoskeletal model was regionally scaled using ground truth MBMC median body segment lengths; this scaled model was used for subsequent inverse kinematics (IK). IK produces a set of joint angles that minimizes the distance between experimental data and musculoskeletal model markers (Figure 2) at each time step. IK was performed with each of the datasets (ground truth MBMC data, filtered PFERD_base data, filtered PFERD-SwRI_Horse data) as the input. Two neck and three tail markers (Figure 2) were excluded from IK due to the available model DOFs. When performing IK with OpenSim for human applications, it is standard practice to down-weight experimental markers for which the experimentalist has low confidence. For this work, if a marker’s average prediction error was greater than half the length of the horse’s third metacarpal bone (~11.5 cm), that marker was excluded during IK due to the low confidence in its predicted value.

The remaining measures were calculated using the IK results. IK marker error is defined as the distance between the model marker and IK input data (Equation (2)); it was determined for each marker at every timepoint (Equation (2)) across each gait period. The RMSE between joint angles calculated using ground truth and network-predicted marker trajectories was determined for each DOF across each gait period.

(2) $$\mathrm{IK}\;\mathrm{Marker}\;\mathrm{Error}\;=\;\sqrt{{\left(X_{IK}-X_{M}\right)}^{2}+{\left(Y_{IK}-Y_{M}\right)}^{2}+{\left(Z_{IK}-Z_{M}\right)}^{2}}$$

where IK = inverse kinematics input data and M = model marker position after running inverse kinematics on the given input dataset

Then, Pearson correlation coefficients (r) and interclass correlation coefficients (ICCs) were determined on a stride-by-stride basis to assess similarity between predictions made by ground truth and network predictions [51]. For this, toe-off of the right front hoof in the ground truth MBMC data were used to identify the start of each stride and joint angles were time normalized across 100 points. Pearson correlation coefficients (r) were computed using *scipy.stats.pearsonr* in Python 3.11.10 [52]. These were interpreted as very high (0.9–1.0), high (0.7–0.9), moderate (0.5–0.7), low (0.3–0.5), and negligible (<0.3) [51]. ICC were estimated based on ICC3 and calculated using *pingouin* [53]; ICC values were interpreted as excellent (>0.9), good (0.75–0.90), moderate (0.5–0.75), and poor (<0.5) [54]. For both r and ICC, the average and range of found values across strides are reported.

## 3. Results

### 3.1. Network Training

**Figure 4:**
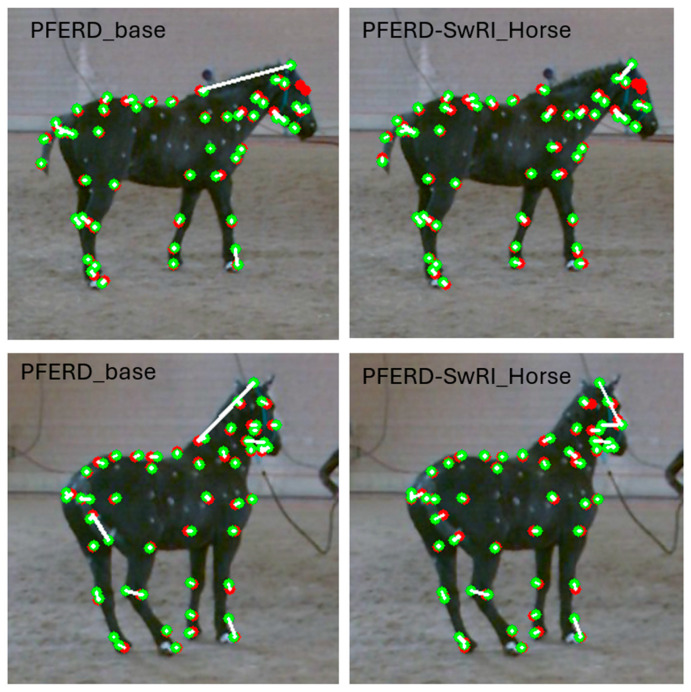
Two-dimensional evaluation examples for the PFERD_base and PFERD-SwRI_Horse networks. The ground truth (green), network prediction (red), and distance between the two (white line) are shown for all skeletal markers for PFERD ID 5. This horse was missing several ground truth (green) markers on the head [29].

Two-dimensional network prediction accuracy was higher for PFERD-SwRI_Horse than PFERD_base (Table 1; Figure 4). Seventy-eight percent of landmarks were within 25% of the radius length (PCKh0.25) to the ground truth location for the PFERD_base network; this value was higher (82%) for the PFERD-SwRI_Horse network (Table 1). The ~30% drop in accuracy between the PCKh0.1 and average PCKh indicates that both networks could place the marker close to the correct location for most samples, but there was noise in the network predictions. The 4-point increase in PCKh between each PFERD-SwRI_Horse and PFERD_base represents an increase in accuracy for the network; the uniform increase across all thresholds indicates a reduction in variance for PFERD-SwRI_Horse network predictions.

### 3.2. Kinematics Results

**Figure 5:**
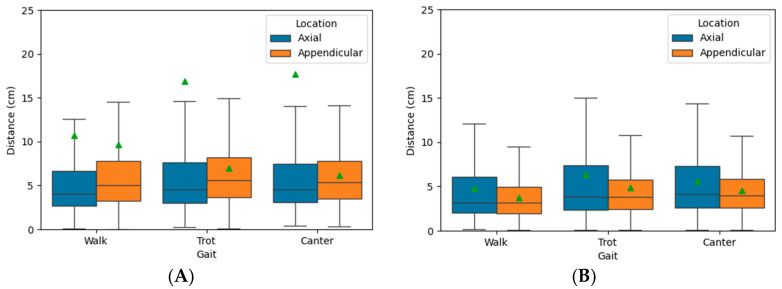
Boxplots of the prediction error for markers predicted using the PFERD_base (**A**) and PFERD-SwRI_Horse (**B**) networks. The median (horizontal line), mean (green triangle), and 1.5× Interquartile Range (whiskers) are shown. The mean, standard deviation, and median values are provided in Table A1.

For the PFERD_base network, median prediction error was lower for axial markers (4.1–4.5 cm) compared with appendicular markers (5.1–5.3 cm) regardless of gait (Figure 5; Table A1). However, the prediction error had high variance and were right skewed (Table A1, Figure 5), likely due to noise among marker predictions. Boxplots of prediction error for each marker are provided in Figure S2 of the Supplementary Materials. At the walk and trot, the poll (marker 1, C_Poll), withers marker (marker 10, C_Back_1), and right tuber coxae (marker 25, R_Pelvis_1) had an average prediction error greater than our exclusion threshold and were not used for IK. At the canter, the poll and withers markers were similarly excluded.

For the PFERD-SwRI_Horse network, the median prediction error was similar for axial (3.2–4.1 cm) and appendicular marker (3.2–3.9 cm) at all gaits (Figure 5; Table A1). Median and average prediction errors were lower than those made by PFERD_base marker set (Table A1). In addition, these prediction errors had lower variance compared with PFERD_base (Table A1), which aligns with the observation of a consistently higher PCKh score for the PFERD-SwRI_Horse network (Table 1). No trends towards higher error at faster gaits were observed. Boxplots of prediction error are provided in Figure S2. The smallest average prediction error was found for the central back markers (marker 11–14, C_Back_2 to _4). The poll marker was excluded from IK at all gaits because it exceeded our threshold.

After performing IK using ground truth marker data, the average IK marker error was approximately 4 cm for axial markers and 3 cm for appendicular markers at all gaits (Table A2; Figure S3). Average IK marker error increased to between 3.5–5 cm when networks predicted marker data were used (Table A2; Figure S4). PFERD-SwRI_Horse network predictions had a lower average IK marker error when compared with PFERD_base (Table A2). A high error was consistently observed for marker 15 (C_Back_6) regardless of the input dataset (Figures S3 and S4).

**Figure 6:**
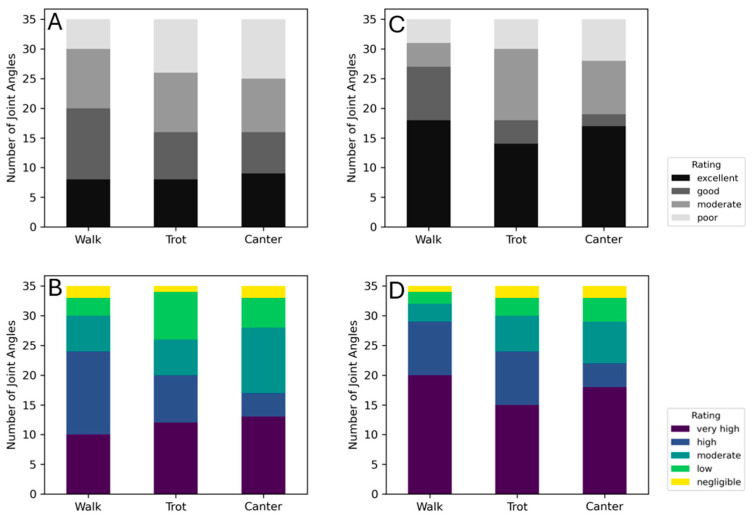
Average interclass correlation coefficient (ICC; panels **A**,**C**) and Pearson correlation coefficient (r; panels **B**,**D**) ratings comparing the 35 joint angles (model DOFs) across validation horse strides. Panels (**A**,**B**) show similarity results comparing the PFERD_base network predictions to ground truth and panels (**C**,**D**) show similarity results comparing PFERD-SwRI_Horse network predictions to ground truth.

**Figure 7:**
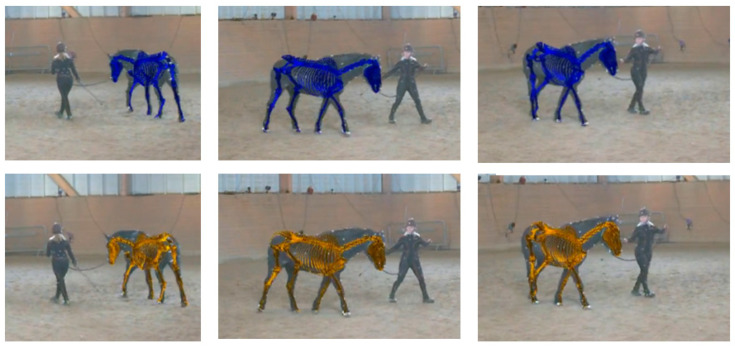
Example overlays of the network-predicted musculoskeletal model (**top row**, blue skeleton, using PFERD-SwRI_Horse) and ground truth data (**bottom row**, orange skeleton) on PFERD Horse ID 5 [29]. The scapula is not shown, so that it is easier to see limb location through the torso.

Joint angle curve similarity (ICC and r) and error (RMSE) metrics indicate that the joint angle predictions made by the network trained on PFERD-SwRI_Horse more closely follow the ground truth IK joint angles than those predicted by the network trained on PFERD_base (Figure 6; Table A4). In general, joint angle RMSE increased distally regardless of the network used to make joint angle predictions (Table A3 and Table A4). However, ICC ratings indicate good to excellent similarity and r ratings indicate moderate to excellent correlation among distal limb predictions (Table A5 and Table A6). Curve shape similarity measures (r and ICC) were lowest for the atlanto-occipital DOF, neck base DOF, and scapulothoracic DOF outside of the sagittal plane (e.g., around local *x* and *y* axes); these angles tended to have a small ROM and high variance in network and ground truth predictions (Figure 7).

Using the PFERD_base network, we observed an RMSE of less than 10° for 24 to 26 of the 35 DOF at the walk, trot, and canter (Table A3; Figure S5), with the highest RMSEs observed for fetlock joint angles. Average ICC scores indicated excellent agreement between network and ground truth joint angles for 10, 12, and 13 DOFs at the walk, trot, and canter, respectively (Figure 6). Similarly, r indicated high to very high correlation to ground truth joint angles among 24, 20, and 17 DOF at the walk, trot, and canter.

**Figure 8:**
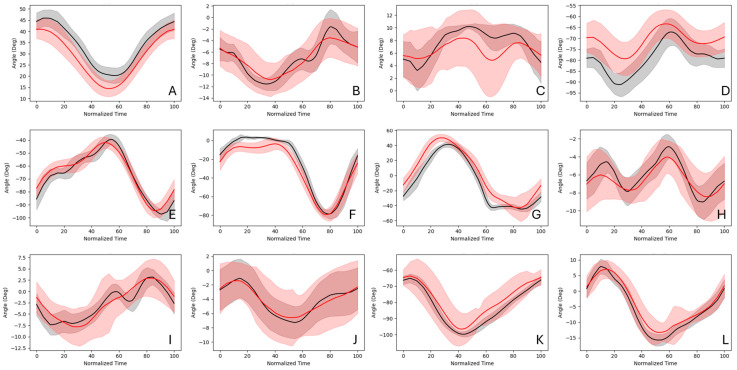

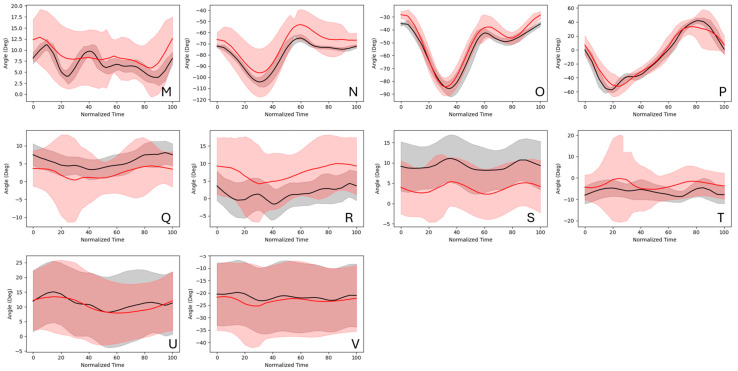
Joint angles calculated using ground truth marker locations (black) and PFERD-SwRI_Horse network predictions (red) across 24 strides of trot. The mean joint angle (solid line) and standard deviation (shaded) are shown for the following left side or axial skeleton joint angles: (**A**) Scapulothoracic (SCT) Z-direction, (**B**) SCT X-direction, (**C**) SCT Y-direction, (**D**) shoulder, (**E**) elbow, (**F**) carpus, (**G**) fore fetlock, (**H**) pelvis Z-direction, (**I**) pelvis X-direction, (**J**) pelvis Y-direction, (**K**) hip flexion (Z-direction), (**L**) hip X-direction, (**M**) hip Y-direction, (**N**) stifle, (**O**) hock, (**P**) hind fetlock, (**Q**) atlanto-occipital X-direction, (**R**) atlanto-occipital Y-direction. (**S**) atlanto-occipital Z-direction, (**T**) neck base X-direction, (**U**) neck back Y-direction, and (**V**) neck base Z-direction.

Joint angle RMSE decreased when the PFERD-SwRI_Horse dataset was used (Table A4) and curve similarity metrics improved (Table A6). Specifically, joint angle RMSE of less than 10° was observed for 32 DOFs, 26 DOFs, and 30 DOFs at the walk, trot, and canter, respectively (Figure 7 and Figure 8; Table A4). ICC ratings were good to excellent for most joint angles and were excellent for 18, 14, and 17 DOFs at the walk, trot, and canter, respectively (Figure 6). Similarly, r indicated high to very high correlation to ground truth joint angles among 29, 21, and 22 DOFs at the walk, trot, and canter, respectively. RMSE improved 16–66% among fetlock angle calculations for all gaits when compared with PFERD_base and curve shape similarity metrics (r and ICC) showed better agreement with ground truth angles.

## 4. Discussion

This study demonstrates that the proposed three-dimensional markerless motion capture pipeline is feasible in horses. Our base neural network (PFERD_base) predicted skeletal landmarks with good accuracy and our pipeline calculated most kinematic trajectories within 10° of the ground truth. To demonstrate that additional data would improve predictions, we also tested our pipeline using a network trained over data from more horses in a wider variety of environments (PFERD-SwRI_Horse). This dataset contained only slightly more images than the base network and were sparsely labeled but included data on more horses in more environments. Decreases in both the mean and variance of marker prediction error indicate that this network provided more accurate and less noisy predictions than the base network. In addition, it resulted in less error and higher curve similarity ratings for joint angle calculated using via inverse kinematics.

Our median prediction errors (3–5 cm) are similar to prediction errors produced by an equine shape and pose model for horses run on the PFERD dataset (3.1 cm) and slightly better than joint-position errors (6.9 ± 0.2 cm) predicted using a different equine dataset [28,29]. We did not see an increase in prediction error at higher speed gaits. This is likely because the frame rate was high enough to capture relatively unblurred images at a slow canter. Our base network performed worst when estimating the poll, withers, tuber coxae, and hoof locations. However, multiple markers on head and spine segments allowed the model to be constrained during IK, even with poor tracking (or excluded markers) in some axial skeleton locations. The combined network performed better on the hooves, tuber coxae, and withers, though not the poll; this was expected, as the SwRI_Horse dataset did not add labeled head anatomy but did contain labeled images of the limbs.

Network predictions and joint angle trajectories could be improved by using more training data and more markers. Additional training data, with more horses in a variety of environments, would improve network robustness and accuracy. Synthetic data (e.g., from VAREN [32]) could supplement MBMC training data [35]. Human MMC systems with more markers and cameras tend to be more accurate [20,35]. Incorporating more markers might reduce errors, especially when segments are partially occluded (e.g., adding a pastern marker to help resolve fetlock motion when the hoof sinks into the footing). However, as the OpenSim IK solver is a global optimization procedure, error in distal segments may have been compounded by errors near to the model’s root (pelvis). Thus, improving overall network performance with additional training data would likely also improve distal limb IK predictions.

We observed a median IK marker error between 3–5 cm; this is higher than the acceptable ~1 cm in human applications [55]. IK errors are less understood in horses, as most studies do not tie marker-based data to a musculoskeletal model when predicting joint angles. However, induced sacral marker error in the range of 3–6 cm has led to misclassification of lameness using sacral vertical displacements [22]. With the PFERD-SwRI_Horse network, we observed an average IK marker error of 3.8 ± 0.7 cm for the sacral marker (Marker #14 in Figure 2; C_Back_5 in Figure S4) across the walk, trot, and canter; so, it may be necessary to reduce IK error in future applications that use this metric to detect gait abnormalities. However, using prior work demonstrates that lameness identification will likely be feasible using time-series data generated by this pipeline, as an equine shape and pose model with joint-position errors averaging 6.9 cm shows promise in predicting lameness [28].

In addition, we observed high to very-high Pearson correlation coefficients (r) and good to excellent interclass correlation coefficients (ICC) on a stride-by-stride basis using PFERD-SwRI_Horse network predictions for most joint angles. These results indicate that, despite prediction and IK marker error, driving IK with network predictions produced kinematic trajectories that are similar in both shape and magnitude to when the model was driven with the ground truth marker-based motion capture data. However, this study used a single validation horse, so future work should include validation over more subjects.

Even when marker locations are precisely known (i.e., there is low prediction error), error in joint angles calculated using IK can arise from poor marker registration (i.e., model marker placement) and model scaling (i.e., model segment dimensions) [55]. Improvements in both marker registration and model scaling would reduce IK marker error and joint angle error and improve curve similarity scores. For instance, separating the fused pelvic body segment to better match equine anatomy may reduce the high IK marker error observed in the sacral region by allowing improved regional scaling and increasing available DOFs. Further, adjusting distances between bony landmarks and model markers using horse body shape data would improve marker registration. This could be achieved by combining our musculoskeletal model with a shape and pose model, like hSMAL [28] or VAREN [32]. As this was a proof-of-concept study, we did not tune marker registration, scaling, or available DOFs. In this study, the model was scaled once with MBMC data to make the joint angle comparisons more directly comparable. In the future, the musculoskeletal model used in the MMC pipeline would be scaled by MMC data.

The musculoskeletal model contains simplifications which could be removed if the training data contained more skeletal markers, as this would allow more motions to be resolved. First, the spine was simplified. Second, the carpus and tarsus, which have multiple centers of rotation, were modeled as a single joint to align with typical MBMC predictions. Third, limb joints were restricted to flexion/extension, as minimal motion occurs in other directions [49,56,57,58,59,60,61]. For example, the fetlock joint shows 62° ROM for flexion/extension, compared with 13° for abduction/adduction and 6° for axial rotation at the walk [59]. Additional non-collinear limb markers in the training data would be needed to resolve motions out of the sagittal plane. Finally, the proximal interphalangeal joint, distal interphalangeal joint, and articulation between the third metacarpal/metatarsal and proximal sesamoid bones were not modeled. A marker on the first phalanx and hoof may allow interphalangeal joint motion to be measured; however, occlusion is likely due to hoof–surface interaction. The distal interphalangeal joint and proximal sesamoid bone interactions are difficult to capture without bone-fixed markers or biplanar videoradiography.

## 5. Conclusions

This study was a proof-of-concept to determine if a full 3D MMC pipeline could be built for horses, in the context of using generated data for lameness identification, performance evaluation, and/or clinical research. We demonstrated that a full 3D MMC system is feasible for horses and observed both low error and good curve similarity ratings when comparing most network-predicted joint angle to ground truth joint angle results. Low to moderate errors in network predictions, joint angle trajectories, and IK marker errors suggest this pipeline could be useful for downstream applications. Future improvements in network training, model scaling, and marker registration could reduce errors in the MMC pipeline.

One of the major advantages of markerless motion capture systems is the reduction in preparation time for captures, as it eliminates the need to instrument the horse. However, this advantage would not be beneficial if this time reduction comes at the cost of significant processing time. Using an NVIDIA 4090 GPU, we measured a processing time of 2 min and 41 s for the PFERD validation horse (an 1800 frame video captured by 10 cameras) to get the network predicted trajectories. This is comparable to the processing times of common marker-based motion capture systems. It took us an additional 5 min to run network predictions through IK, providing a total time from video input to kinematic data of less than 10 min. When these data were collected, it took ~2 h to instrument each horse (over 100 markers) [29] and would require additional time to process, so the proposed methodology offers a clear improvement in time and scalability.

A second advantage of a full 3D MMC system is the ease with which full body kinematics are produced. Most equine studies have focused on collecting data from a limited number of body regions, this is likely due to the difficulties associated with collecting full-body kinematic data using existing systems [29]. Similarly, most existing equine MMC systems focus on reporting data for a limited number of body regions or metrics derived from this region [16,17,23,24,25,26,27]. The ease of use and scalability improvements offered by a full-body MMC pipeline allow for a better understanding of full body motions, larger study sizes, and, perhaps, more metrics that can be used to predict horse health. In human biomechanics, MMC has made the advent of personalized biomechanical analysis feasible, and many associated methodologies could translate to the equine space.

In our MMC pipeline, musculoskeletal model movement (resulting from IK) can be post-processed to produce any kinematic metric of interest that is tied to skeletal movement and feasible with model DOF restrictions. Using lameness detection as an example, stride characteristics (e.g., swing/stance variation) and vertical displacement asymmetries of the sacrum, withers, poll, and pelvis are used clinically to identify lameness [7,16,17,18,22]. These measures can be determined from the IK output. However, lameness identification by this MMC pipeline would need to be directly compared with gold-standard lameness detection systems to determine if refinements need to be made before the MMC pipeline could be used for clinical diagnoses.

The methodology developed in this work represents an advancement in technologies available for equine kinematic analysis. One advantage of using a musculoskeletal model, over a 3D shape and pose model, is that motion can be easily constrained to physiologic limits. Additionally, future implementations can leverage the model’s ability to predict the kinetic components of gait (e.g., joint reaction and soft tissue forces) using simulated ground–hoof interaction or traditional ground-reaction force measures. Musculoskeletal loading, along with motion, can cause and indicate the presence of injury and affect athletic performance; so, kinetic and kinematic data may be useful for lameness prediction and performance feedback.

The current study presents a methodology and preliminary validation of a 3D markerless motion capture pipeline capable of quantifying full-body equine kinematics for a variety of downstream applications—including lameness identification, athletic performance monitoring, and horse–rider interactions. Our preliminary validation provided high similarity between MMC- and MBMC-predicted joint angles produced by the full pipeline. These findings suggest that the system will be usable for downstream applications; however, future work needs to be done to determine what refinements need to be made for any given use case. Importantly, this markerless approach offers scalability and flexibility to produce full-body time-series data which can be leveraged when producing any diagnostic output of interest.

## Figures and Tables

**Table 1:** Network training results on the network testing split (PFERD ID 5). PCKhA indicates the percent of skeletal markers identified within A × 100% of the threshold distance (e.g., PCKh0.1 indicates the percent within 10% of the threshold to the true location).

Model	PCKh0.1	PCKh0.25	PCKh0.5	PCKh1.0	Average PCKh
PFERD_base	47.4	78.6	88.2	92.1	76.5
PFERD-SwRI_Horse	49.8	82.6	92.4	95.7	80.1

## Data Availability

A majority of the data used in this study are available in the Poses for Equine Research Dataset at DOI: 10.1038/s41597-024-03312-1 (dataset link: https://doi.org/10.7910/DVN/2EXONE), A Novel Dataset for Keypoint Detection of Quadruped Animals at 10.48550/arXiv.2108.13958 (dataset link: https://github.com/prinik/AwA-Pose. accessed on 17 April 2025), Equine Kinematic Gait Analysis Using Stereo Videography and Deep Learning: Stride Length and Stance Duration Estimation at 10.13031/ja.15386 (dataset link: https://github.com/NarimanNiknejad/DLC_Horse. accessed on 17 April 2025), and AP-10K at 10.48550/arXiv.2108.12617 (dataset link: https://github.com/AlexTheBad/AP-10K. accessed on 17 April 2025). Remaining data were sourced from 3rd parties and are described in 10.3390/ani14162410, 10.3390/ani13132122, and 10.1111/evj.12113; requests for access to these datasets should be directed to the relevant authors. The musculoskeletal model referred in DOI: https://doi.org/10.1093/icb/icae095 is available at https://simtk.org/projects/shadowfax. accessed on 17 April 2025.

## Supplementary Materials

The following supporting information can be downloaded at https://www.mdpi.com/article/10.3390/ani15152281/s1, Figure S1: Example time traces for left and right carpus and fore fetlock joint flexion/extension angles across 24 strides of the trot; Figure S2: Prediction Error at the walk, trot, and canter for the network trained on PFERD_base and on PFERD_SwRI-Horse; Figure S3: Inverse Kinematics Marker Error using ground truth data for the validation horse at the walk, trot, and canter.; Figure S4: Inverse Kinematics Marker Error at the walk (top), trot (center), and canter (bottom) using predicted data for the networks trained on PFERD_base and PFERD_SwRI-Horse.; Figure S5: Examples of joint angles calculated using ground-truth marker locations compared to those calculated using PFERD_base network predictions across 24 strides of trot.

## Appendix A

**Table A1:** The average, standard deviation, and median prediction error at each gait for axial and appendicular markers. Results were calculated over 14 strides of walk, 24 strides of trot, and 8 strides of canter for the pipeline validation horse.

	PFERD_Base Prediction Error (cm) Average ± Standard Deviation (Median)		PFERD-SwRI_Horse Prediction Error (cm) Average ± Standard Deviation (Median)	
	Axial Markers	Appendicular Markers	Axial Markers	Appendicular Markers
Walk	10.7 ± 156.1 (4.1)	9.7 ± 93.7 (5.1)	4.8 ± 4.4 (3.2)	3.7 ± 2.4 (3.2)
Trot	16.9 ± 207.5 (4.6)	6.9 ± 20.7 (5.6)	6.4 ± 24.8 (3.8)	4.9 ± 5.4 (3.8)
Canter	17.7 ± 110.8 (4.5)	6.2 ± 4.1 (5.3)	5.6 ± 4.6 (4.1)	4.5 ± 2.7 (3.9)

**Table A2:** The average, standard deviation, and median values for IK marker error at each timepoint for axial and appendicular markers. Results were calculated over 14 strides of walk, 24 strides of trot, and 8 strides of canter for the pipeline validation horse.

	Ground Truth IK Marker Error Average ± Standard Deviation (Median)		PFERD_Base IK Marker Error Average ± Standard Deviation (Median)		PFERD-SwRI_Horse IK Marker Error Average ± Standard Deviation (Median)	
	Axial Markers	Appendicular Markers	Axial Markers	Appendicular Markers	Axial Markers	Appendicular Markers
Walk	4.2 ± 2.4 (3.8)	3.0 ± 2.0 (2.7)	4.9 ± 3.2 (4.2)	4.3 ± 2.2 (4.3)	4.7 ± 3 (4.1)	3.5 ± 1.9 (3.4)
Trot	4.2 ± 2.3 (3.9)	3.0 ± 2.0 (2.6)	4.9 ± 3.1 (4.3)	4.6 ± 2.4 (4.4)	5 ± 3.3 (4.3)	4.1 ± 3.6 (3.8)
Canter	4.1 ± 2.2 (3.8)	2.9 ± 1.9 (2.6)	4.7 ± 2.9 (4.0)	4.3 ± 2.1 (4.2)	4.9 ± 3.0 (4.2)	3.6 ± 1.9 (3.5)

**Table A3:** Joint angle RMSE (in degrees) comparing joint angles calculated via IK with ground truth and PFERD_base network predictions. Rotations around the joint local *x*, *y*, and *z* axes are indicated by Rx, Ry, and Rz, respectively. For all limb angles, flexion/extension is rotation about the local *z*-axis (Rz).

	Angle	Walk	Trot	Canter
Forelimb Joint Angles	SCT; left (Rz)	4.9	3.8	3.9
	SCT; right (Rz)	3.9	4.6	4.4
	SCT; left (Rx)	2.0	3.9	3.1
	SCT; right (Rx)	3.5	5.6	2.4
	SCT; left (Ry)	6.5	8.1	6.5
	SCT; right (Ry)	3.7	5.7	4.2
	Shoulder; left (Rz)	4.5	5.9	6.7
	Shoulder; right (Rz)	4.8	8.6	6.0
	Elbow; left (Rz)	7.0	7.0	7.7
	Elbow; right (Rz)	6.3	8.1	6.9
	Carpus; left (Rz)	12.5	13.5	12.7
	Carpus; right (Rz)	12.3	11.1	11.3
	Fore fetlock; left (Rz)	36.2	38.4	41.1
	Fore fetlock; right (Rz)	32.8	36.1	27.6
Hindlimb Joint Angles	Hip; left (Rz)	7.0	6.2	4.8
	Hip; right (Rz)	9.8	10.2	10.4
	Hip; left (Rx)	3.3	5.8	4.6
	Hip; right (Rx)	4.3	7.3	6.0
	Hip; left (Ry)	4.8	4.9	4.1
	Hip; right (Ry)	5.2	6.9	8.7
	Stifle; left (Rz)	10.7	9.6	8.7
	Stifle; right (Rz)	13.1	14.1	13.2
	Hock; left (Rz)	10.9	8.3	6.4
	Hock; right (Rz)	12.4	14.9	15.1
	Hind fetlock; left (Rz)	13.6	17.6	13.4
	Hind fetlock; right (Rz)	21.9	24.9	21.6
Other Joint Angles	Atlanto-occipital (Rx)	3.9	4.5	4.5
	Atlanto-occipital (Ry)	4.5	7.4	9.0
	Atlanto-occipital (Rz)	2.1	3.5	3.9
	Neck base (Rx)	3.1	5.9	3.8
	Neck base (Ry)	2.8	3.5	3.2
	Neck base (Rz)	2.5	2.7	2.2
	Pelvic; (Rx)	3.1	5.9	3.8
	Pelvic; (Ry)	2.8	3.5	3.2
	Pelvic; (Rz)	2.5	2.7	2.2

**Table A4:** RMSE (degrees) comparing joint angles calculated via IK with ground truth and PFERD-SwRI_Horse network predictions. Rotations around the joint local *x*, *y*, and *z* axes are indicated by Rx, Ry, and Rz, respectively. For all limb angles, flexion/extension is rotation about the local *z*-axis (Rz).

	Angle Name	Walk	Trot	Canter
Forelimb Joint Angles	SCT; left (Rz)	5.5	6.6	4.2
	SCT; right (Rz)	3.5	4.3	4.6
	SCT; left (Rx)	2.1	3.3	2.6
	SCT; right (Rx)	2.2	5.1	2.7
	SCT; left (Ry)	2.2	4.4	2.8
	SCT; right (Ry)	4.1	4.7	5.0
	Shoulder; left (Rz)	7.5	10.6	7.7
	Shoulder; right (Rz)	8.6	10.3	11.0
	Elbow; left (Rz)	4.6	6.8	7.8
	Elbow; right (Rz)	8.4	10.0	9.3
	Carpus; left (Rz)	10.2	9.6	9.1
	Carpus; right (Rz)	4.7	9.9	7.4
	Fore Fetlock; left (Rz)	15.0	15.3	13.6
	Fore Fetlock; right (Rz)	16.6	28.5	23.8
Hindlimb Joint Angles	Hip; left (Rz)	4.2	9.9	3.7
	Hip; right (Rz)	2.7	4.9	3.6
	Hip; left (Rx)	1.9	4.2	2.6
	Hip; right (Rx)	2.3	5.0	4.5
	Hip; left (Ry)	4.7	6.4	3.7
	Hip; right (Ry)	3.3	7.1	6.2
	Stifle; left (Rz)	9.1	18.0	7.8
	Stifle; right (Rz)	3.8	8.8	4.1
	Hock; left (Rz)	5.9	7.8	6.5
	Hock; right (Rz)	6.1	7.9	7.4
	Hind fetlock; left (Rz)	8.0	16.5	15.3
	Hind fetlock; right (Rz)	7.2	14.7	12.2
Other Joint Angle	Atlanto-occipital (Rx)	3.7	7.2	3.5
	Atlanto-occipital (Ry)	6.7	9.3	9.1
	Atlanto-occipital (Rz)	5.2	6.5	5.2
	Neck base (Rx)	3.9	10.8	5.4
	Neck base (Ry)	2.8	5.0	4.5
	Neck base (Rz)	1.6	4.8	1.8
	Pelvic; (Rx)	2.8	4.4	3.3
	Pelvic; (Ry)	2.1	2.9	2.7
	Pelvic; (Rz)	1.4	2.4	1.5

**Table A5:** Curve similarity measures (average [minimum–maximum]) for joint angles calculated per stride using ground truth and PERFD_base IK results. Rotations around the joint local *x*, *y*, and *z* axes are indicated by Rx, Ry, and Rz, respectively. For all limb angles, flexion/extension is rotation about the local *z*-axis (Rz).

	Angle	Pearson Correlation Coefficient			Interclass Correlation Coefficient		
		Walk	Trot	Canter	Walk	Trot	Canter
Forelimb Joint Angles	SCT; left (Rz)	0.95 [0.91–0.98]	0.96 [0.88–1.00]	0.95 [0.87–0.98]	0.87 [0.75–0.94]	0.94 [0.80–0.99]	0.89 [0.77–0.94]
	SCT; right (Rz)	0.96 [0.85–0.99]	0.96 [0.87–1.00]	0.92 [0.86–0.96]	0.93 [0.77–0.98]	0.96 [0.87–0.99]	0.91 [0.81–0.96]
	SCT; left (Rx)	0.78 [0.02–0.96]	0.72 [0.13–0.96]	0.32 [-0.22–0.77]	0.76 [0.02–0.96]	0.67 [0.12–0.96]	0.31 [-0.21–0.77]
	SCT; right (Rx)	0.58 [−0.29–0.92]	0.64 [0.04–0.91]	0.18 [−0.26–0.89]	0.55 [−0.28–0.86]	0.58 [0.04–0.87]	0.18 [−0.24–0.86]
	SCT; left (Ry)	0.19 [−0.21–0.80]	0.30 [−0.80–0.93]	0.31 [−0.01–0.69]	0.10 [−0.17–0.52]	0.25 [−0.76–0.90]	0.21 [−0.01–0.66]
	SCT; right (Ry)	0.49 [−0.30–0.83]	0.23 [−0.47–0.83]	0.33 [−0.18–0.68]	0.46 [−0.30–0.82]	0.22 [−0.42–0.83]	0.31 [−0.17–0.68]
	Shoulder; left (Rz)	0.83 [0.69–0.96]	0.80 [0.09–0.98]	0.61 [0.22–0.88]	0.76 [0.49–0.92]	0.77 [0.09–0.93]	0.53 [0.21–0.82]
	Shoulder; right (Rz)	0.80 [0.56–0.91]	0.69 [−0.57–0.92]	0.63 [0.39–0.92]	0.77 [0.51–0.91]	0.65 [−0.52–0.90]	0.61 [0.39–0.90]
	Elbow; left (Rz)	0.98 [0.96–0.99]	0.96 [0.92–0.98]	0.95 [0.93–0.98]	0.95 [0.90–0.98]	0.95 [0.84–0.97]	0.93 [0.88–0.97]
	Elbow; right (Rz)	0.93 [0.74–0.98]	0.93 [0.79–0.99]	0.92 [0.80–0.96]	0.93 [0.73–0.98]	0.92 [0.74–0.99]	0.92 [0.76–0.96]
	Carpus; left (Rz)	0.84 [0.62–0.98]	0.91 [0.70–0.99]	0.90 [0.80–0.98]	0.83 [0.60–0.98]	0.89 [0.62–0.99]	0.90 [0.77–0.98]
	Carpus; right (Rz)	0.93 [0.77–0.99]	0.95 [0.75–1.00]	0.97 [0.96–0.98]	0.92 [0.77–0.99]	0.94 [0.70–1.00]	0.95 [0.89–0.97]
	Fore fetlock; left (Rz)	0.82 [0.67–0.89]	0.82 [-0.75–0.98]	0.65 [0.26–0.92]	0.68 [0.55–0.79]	0.77 [-0.63–0.95]	0.62 [0.24–0.92]
	Fore fetlock; right (Rz)	0.64 [0.09–0.96]	0.45 [-0.69–0.97]	0.82 [0.63–0.95]	0.60 [0.09–0.94]	0.42 [-0.69–0.95]	0.78 [0.61–0.95]
Hindlimb Joint Angles	Hip; left (Rz)	0.95 [0.71–0.99]	0.97 [0.86–1.00]	0.98 [0.98–0.99]	0.93 [0.60–0.99]	0.97 [0.86–1.00]	0.98 [0.98–0.99]
	Hip; right (Rz)	0.97 [0.90–0.99]	0.96 [0.88–1.00]	0.92 [0.85–0.98]	0.93 [0.81–0.99]	0.95 [0.88–0.99]	0.91 [0.85–0.98]
	Hip; left (Rx)	0.93 [0.83–0.98]	0.80 [-0.31–0.99]	0.83 [0.58–0.94]	0.92 [0.82–0.98]	0.77 [−0.19–0.99]	0.77 [0.56–0.92]
	Hip; right (Rx)	0.81 [0.47–0.96]	0.53 [-0.67–0.88]	0.53 [0.16–0.79]	0.79 [0.46–0.95]	0.51 [−0.47–0.84]	0.46 [0.16–0.79]
	Hip; left (Ry)	0.57 [−0.11–0.90]	0.39 [−0.48–0.77]	0.61 [−0.45–0.92]	0.55 [−0.11–0.90]	0.35 [−0.36–0.74]	0.59 [−0.45–0.91]
	Hip; right (Ry)	0.22 [−0.35–0.54]	0.46 [−0.20–0.89]	0.52 [−0.15–0.86]	0.20 [−0.32–0.53]	0.41 [−0.20–0.88]	0.43 [−0.12–0.71]
	Stifle; left (Rz)	0.78 [0.38–0.94]	0.92 [0.60–0.99]	0.92 [0.73–0.98]	0.74 [0.35–0.93]	0.88 [0.59–0.99]	0.85 [0.61–0.94]
	Stifle; right (Rz)	0.91 [0.80–0.98]	0.85 [0.64–0.98]	0.60 [−0.08–0.89]	0.90 [0.78–0.97]	0.81 [0.56–0.98]	0.57 [−0.07–0.88]
	Hock; left (Rz)	0.68 [0.30–0.93]	0.94 [0.76–0.98]	0.91 [0.81–0.98]	0.66 [0.30–0.92]	0.93 [0.76–0.98]	0.90 [0.76–0.96]
	Hock; right (Rz)	0.71 [0.38–0.98]	0.74 [0.24–0.98]	0.63 [−0.28–0.97]	0.69 [0.38–0.97]	0.71 [0.23–0.97]	0.60 [−0.28–0.97]
	Hind fetlock; left (Rz)	0.90 [0.72–0.97]	0.91 [0.74–0.98]	0.96 [0.93–0.98]	0.89 [0.72–0.97]	0.90 [0.73–0.98]	0.95 [0.92–0.97]
	Hind fetlock; right (Rz)	0.77 [0.61–0.98]	0.78 [0.22–0.97]	0.82 [0.08–0.99]	0.76 [0.61–0.98]	0.74 [0.22–0.96]	0.81 [0.08–0.99]
Other Joint Angles	Atlanto-occipital (Rx)	0.37 [−0.44–0.72]	0.43 [−0.47–0.94]	0.55 [−0.37–0.96]	0.33 [−0.44–0.71]	0.38 [−0.37–0.88]	0.44 [−0.33–0.73]
	Atlanto-occipital (Ry)	0.48 [−0.07–0.95]	0.41 [−0.62–0.92]	0.28 [−0.38–0.71]	0.41 [−0.07–0.92]	0.36 [−0.49–0.91]	0.22 [−0.22–0.51]
	Atlanto-occipital (Rz)	0.85 [0.68–0.99]	0.72 [0.19–0.99]	0.72 [−0.40–0.99]	0.82 [0.68–0.99]	0.66 [0.16–0.98]	0.71 [−0.33–0.97]
	Neck base (Rx)	0.64 [0.13–0.91]	0.31 −0.42–0.89]	0.58 [−0.06–0.91]	0.59 [0.11–0.89]	0.30 [−0.33–0.89]	0.53 [−0.04–0.90]
	Neck base (Ry)	0.83 [0.01–0.99]	0.56 [−0.86–1.00]	0.61 [−0.46–0.95]	0.81 [0.01–0.99]	0.53 [−0.83–0.99]	0.56 [−0.36–0.81]
	Neck base (Rz)	0.95 [0.81–1.00]	0.90 [0.73–1.00]	0.93 [0.79–0.98]	0.95 [0.81–1.00]	0.87 [0.44–0.99]	0.92 [0.76–0.98]
	Pelvic; (Rx)	0.86 [0.74–0.96]	0.64 [−0.48–0.94]	0.44 [0.09–0.74]	0.84 [0.72–0.96]	0.58 [-0.47–0.94]	0.41 [0.08–0.71]
	Pelvic; (Ry)	0.72 [−0.01–0.91]	0.47 [−0.40–0.95]	0.45 [−0.05–0.87]	0.64 [−0.01–0.89]	0.43 [−0.40–0.85]	0.42 [−0.04–0.81]
	Pelvic; (Rz)	0.63 [0.01–0.79]	0.67 [−0.14–0.92]	0.97 [0.93–0.99]	0.59 [0.01–0.77]	0.58 [−0.14–0.86]	0.95 [0.88–0.98]

**Table A6:** Curve similarity measures (average [minimum–maximum]) for joint angles calculated per stride using ground truth and PERFD_SwRI_Horse IK results. Rotations around the joint local *x*, *y*, and *z* axes are indicated by Rx, Ry, and Rz, respectively. For all limb angles, flexion/extension is rotation about the local z-axis (Rz).

	Angle	Pearson Correlation Coefficient			Interclass Correlation Coefficient		
		Walk	Trot	Canter	Walk	Trot	Canter
Forelimb Joint Angles	SCT; left (Rz)	0.97 [0.86–0.99]	0.95 [0.65–0.99]	0.97 [0.90–0.99]	0.96 [0.83–0.99]	0.94 [0.63–0.99]	0.96 [0.89–0.99]
	SCT; right (Rz)	0.97 [0.93–0.99]	0.95 [0.67–0.99]	0.97 [0.90–0.98]	0.97 [0.92–0.99]	0.93 [0.67–0.99]	0.95 [0.89–0.97]
	SCT; left (Rx)	0.77 [0.36–0.97]	0.73 [0.06–0.96]	0.43 [−0.20–0.70]	0.75 [0.33–0.97]	0.68 [0.06–0.96]	0.38 [−0.18–0.70]
	SCT; right (Rx)	0.82 [0.61–0.95]	0.57 [−0.40–0.95]	−0.03 [−0.50–0.63]	0.79 [0.50–0.93]	0.51 [−0.39–0.94]	−0.01 [−0.42–0.50]
	SCT; left (Ry)	0.46 [−0.22–0.96]	0.44 [−0.64–0.94]	0.28 [−0.06–0.80]	0.32 [−0.22–0.96]	0.43 [−0.41–0.93]	0.23 [−0.06–0.79]
	SCT; right (Ry)	0.78 [0.51–0.93]	0.68 [0.14–0.96]	0.39 [−0.26–0.93]	0.74 [0.48–0.93]	0.62 [0.13–0.95]	0.35 [−0.26–0.90]
	Shoulder; left (Rz)	0.91 [0.66–0.97]	0.77 [0.04–0.97]	0.64 [0.32–0.78]	0.88 [0.54–0.96]	0.75 [0.04–0.96]	0.57 [0.23–0.75]
	Shoulder; right (Rz)	0.80 [0.60–0.91]	0.59 [−0.09–0.95]	0.66 [−0.04–0.88]	0.78 [0.51–0.91]	0.56 [v0.09–0.94]	0.65 [−0.04–0.87]
	Elbow; left (Rz)	0.98 [0.95–1.00]	0.96 [0.91–0.98]	0.94 [0.79–0.97]	0.97 [0.92–0.99]	0.95 [0.91–0.97]	0.92 [0.74–0.97]
	Elbow; right (Rz)	0.98 [0.95–0.99]	0.93 [0.78–0.99]	0.93 [0.89–0.97]	0.98 [0.92–0.99]	0.93 [0.75–0.99]	0.92 [0.88–0.97]
	Carpus; left (Rz)	0.96 [0.82–1.00]	0.98 [0.85–1.00]	0.98 [0.98–0.99]	0.96 [0.82–1.00]	0.97 [0.85–0.99]	0.97 [0.89–0.98]
	Carpus; right (Rz)	0.99 [0.98–1.00]	0.95 [0.34–1.00]	0.98 [0.96–0.99]	0.99 [0.98–1.00]	0.95 [0.33–0.99]	0.98 [0.96–0.99]
	Fore fetlock; left (Rz)	0.96 [0.86–0.98]	0.97 [0.86–0.99]	0.97 [0.95–0.98]	0.96 [0.86–0.98]	0.96 [0.83–0.99]	0.96 [0.95–0.98]
	Fore fetlock; right (Rz)	0.92 [0.66–0.97]	0.67 [-0.65–0.98]	0.78 [0.37–0.97]	0.90 [0.65–0.96]	0.65 [−0.65–0.96]	0.72 [0.30–0.93]
Hindlimb Joint Angles	Hip; left (Rz)	0.98 [0.95–0.99]	0.94 [0.51–1.00]	0.98 [0.97–0.99]	0.97 [0.95–0.99]	0.93 [0.40–0.99]	0.98 [0.94–0.99]
	Hip; right (Rz)	0.98 [0.94–1.00]	0.98 [0.95–1.00]	0.97 [0.91–0.99]	0.98 [0.90–0.99]	0.97 [0.72–1.00]	0.97 [0.91–0.98]
	Hip; left (Rx)	0.97 [0.94–0.99]	0.90 [0.06–1.00]	0.91 [0.77–0.96]	0.97 [0.94–0.99]	0.89 [0.06–0.99]	0.89 [0.74–0.96]
	Hip; right (Rx)	0.95 [0.88–0.98]	0.73 [−0.40–0.98]	0.76 [0.22–0.96]	0.94 [0.85–0.98]	0.71 [−0.35–0.98]	0.72 [0.20–0.95]
	Hip; left (Ry)	0.62 [0.07–0.84]	0.47 [−0.59–0.80]	0.77 [0.52–0.91]	0.59 [0.07–0.84]	0.43 [−0.43–0.75]	0.75 [0.52–0.89]
	Hip; right (Ry)	0.60 [0.18–0.81]	0.65 [−0.04–0.96]	0.64 [−0.34–0.86]	0.59 [0.17–0.81]	0.62 [−0.03–0.96]	0.64 [−0.22–0.86]
	Stifle; left (Rz)	0.96 [0.89–0.98]	0.90 [−0.71–0.99]	0.95 [0.93–0.99]	0.92 [0.85–0.98]	0.87 [−0.46–0.99]	0.94 [0.91–0.99]
	Stifle; right (Rz)	0.97 [0.91–0.99]	0.94 [0.41–0.99]	0.95 [0.89–0.99]	0.96 [0.91–0.99]	0.92 [0.23–0.99]	0.93 [0.84–0.98]
	Hock; left (Rz)	0.95 [0.91–0.99]	0.96 [0.63–0.99]	0.94 [0.91–0.97]	0.94 [0.90–0.99]	0.95 [0.62–0.99]	0.94 [0.91–0.96]
	Hock; right (Rz)	0.97 [0.93–0.99]	0.96 [0.84–0.99]	0.96 [0.94–0.98]	0.97 [0.93–0.99]	0.96 [0.72–0.99]	0.95 [0.93–0.98]
	Hind fetlock; left (Rz)	0.97 [0.84–0.99]	0.91 [0.04–0.99]	0.94 [0.77–0.98]	0.97 [0.84–0.99]	0.90 [0.04–0.99]	0.93 [0.75–0.98]
	Hind fetlock; right (Rz)	0.98 [0.95–0.99]	0.95 [0.75–0.99]	0.95 [0.85–0.99]	0.97 [0.94–0.99]	0.94 [0.75–0.99]	0.94 [0.84–0.99]
Other Joint Angles	Atlanto-occipital (Rx)	0.24 [−0.70–0.90]	0.48 [−0.56–0.97]	0.69 [0.04–0.99]	0.22 [-0.59–0.85]	0.47 [−0.25–0.97]	0.64 [0.03–0.96]
	Atlanto-occipital (Ry)	0.45 [−0.24–0.96]	0.27 [−0.71–0.97]	0.38 [−0.19–0.62]	0.37 [−0.17–0.94]	0.24 [−0.60–0.92]	0.32 [−0.16–0.56]
	Atlanto-occipital (Rz)	0.83 [0.61–0.98]	0.74 [0.09–0.97]	0.72 [−0.30–0.99]	0.81 [0.60–0.97]	0.68 [0.09–0.97]	0.71 [−0.29–0.99]
	Neck base (Rx)	0.53 [0.11–0.93]	0.16 [−0.63–0.92]	0.52 [−0.59–0.94]	0.43 [0.11–0.84]	0.15 [−0.63–0.92]	0.47 [−0.55–0.88]
	Neck base (Ry)	0.81 [−0.33–0.99]	0.68 [−0.05–0.99]	0.63 [−0.05–0.93]	0.79 [−0.33–0.99]	0.62 [−0.03–0.98]	0.51 [−0.05–0.86]
	Neck base (Rz)	0.97 [0.91–1.00]	0.85 [−0.37–1.00]	0.92 [0.77–0.98]	0.96 [0.89–1.00]	0.81 [−0.13–0.99]	0.90 [0.75–0.97]
	Pelvic; (Rx)	0.86 [0.69–0.95]	0.73 [−0.53–0.98]	0.63 [0.19–0.93]	0.83 [0.66–0.94]	0.68 [−0.39–0.98]	0.61 [0.18–0.92]
	Pelvic; (Ry)	0.86 [0.56–0.97]	0.77 [0.21–0.98]	0.38 [−0.19–0.95]	0.83 [0.55–0.96]	0.70 [0.17–0.96]	0.36 [−0.17–0.87]
	Pelvic; (Rz)	0.78 [0.64–0.93]	0.80 [−0.44–0.97]	0.97 [0.94–0.99]	0.76 [0.61–0.91]	0.77 [−0.31–0.96]	0.96 [0.93–0.99]
