# Supplementary material for: A Markerless Approach for Full-Body Biomechanics of Horses
Source: Animals (Basel). 2025 Aug 5;15(15):2281. doi: 10.3390/ani15152281 (PMC12345546; doi:10.3390/ani15152281)
Supplement: Supplementary file 1 [file animals-15-02281-s001.zip › animals-3618164-supplementary.pdf]

Supplemental Material

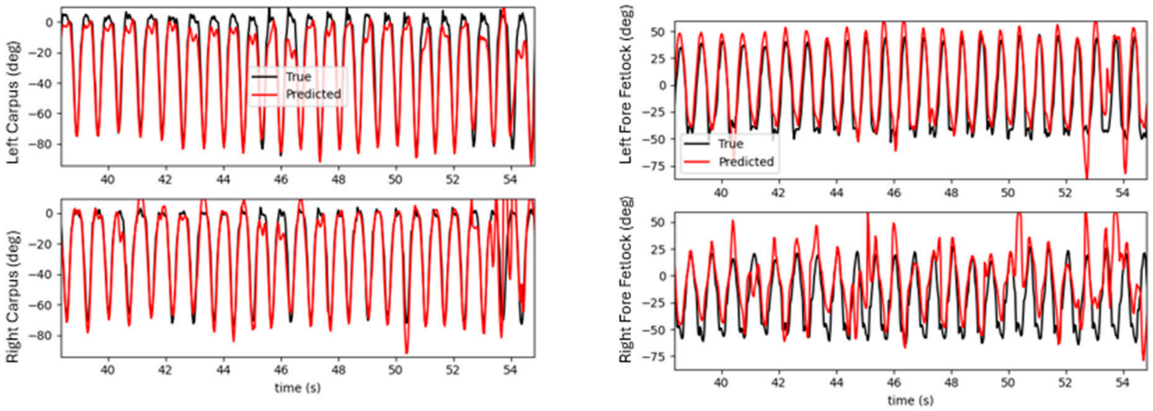

Figure S1: Example time traces for left and right carpus and fore fetlock joint flexion/extension angles across 24 strides of the trot.

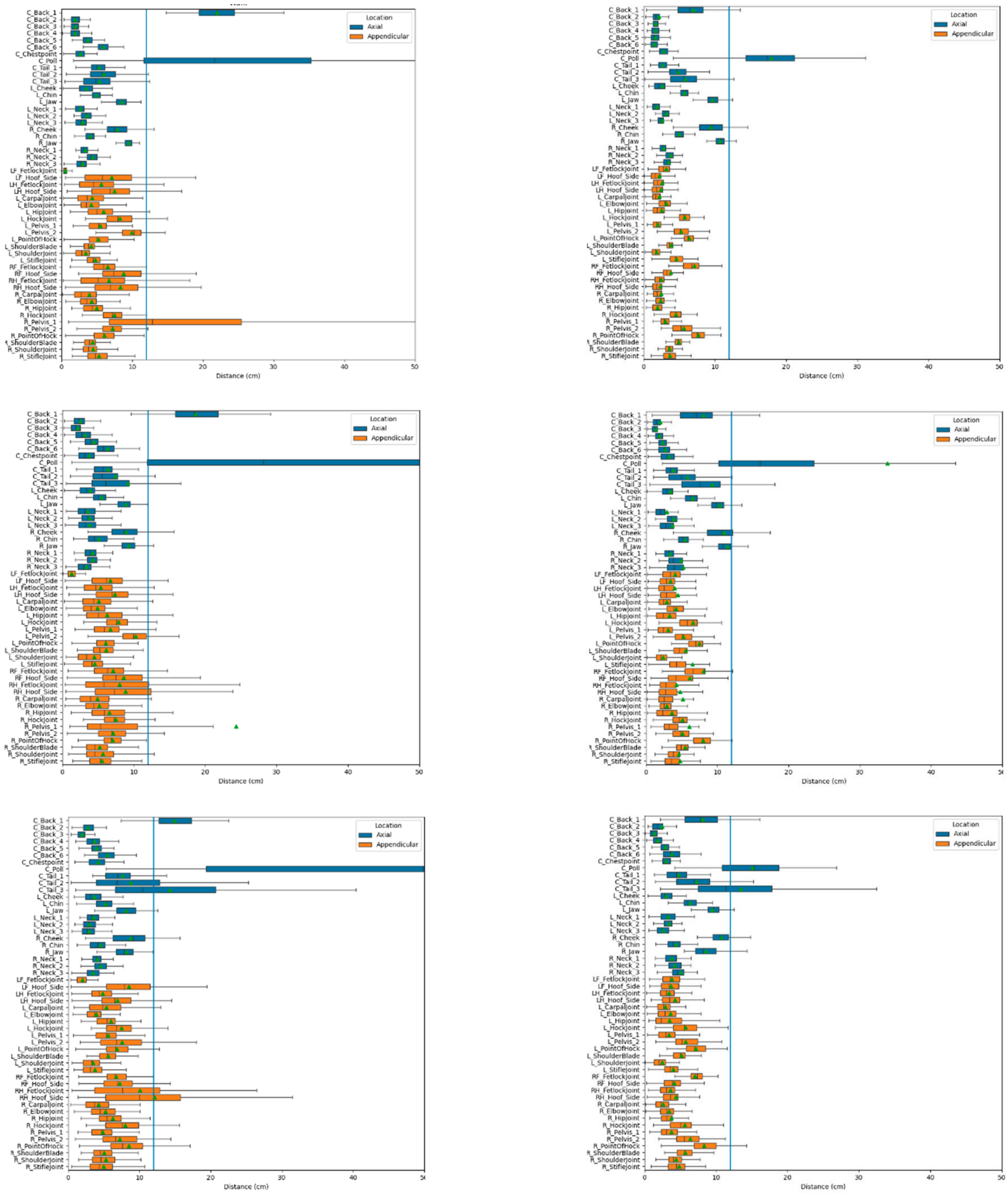

Figure S2: Prediction Error at the walk (top), trot (center), and canter (bottom) for the network trained on PFERD_base (Left) and on PFERD-SwRI_Horse (Right). A blue horizontal line is drawn at the length of one half of the horse's third metacarpal bone. Boxplots show the mean (green triangle), median (black line in boxplot), and 1.5 times the Interquartile Range (whisker limits).

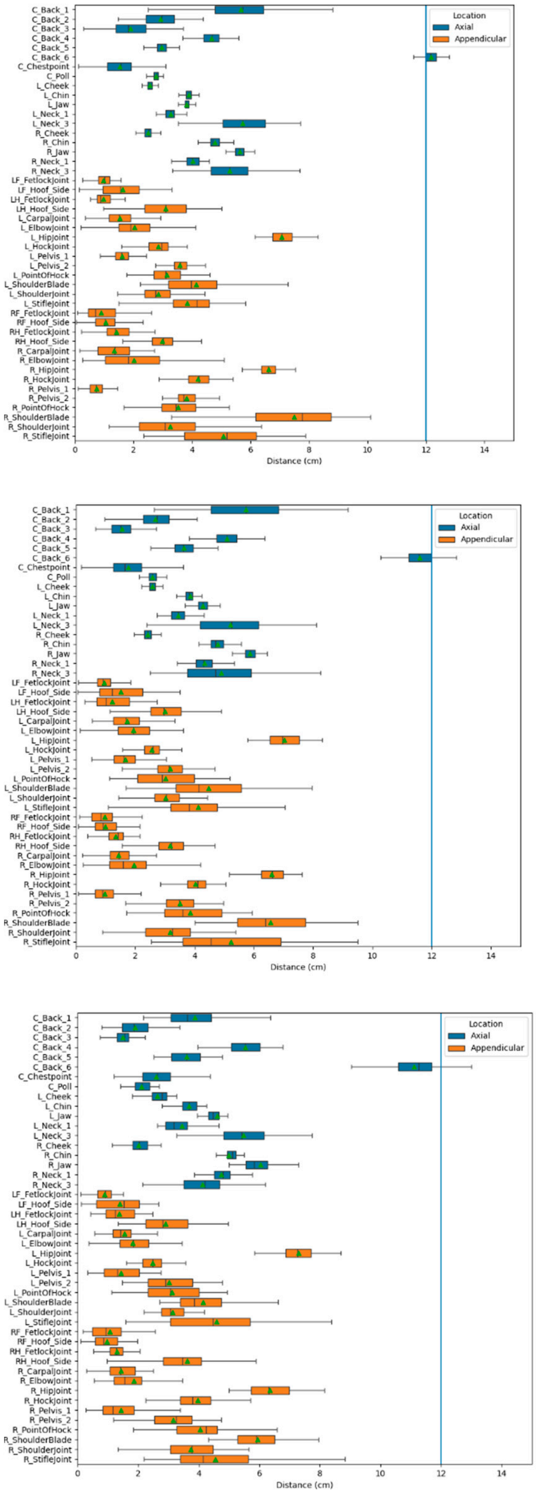

Figure S3: Inverse Kinematics Marker Error using ground truth data for the validation horse at the walk (top), trot (center), and canter (bottom). A blue horizontal line is drawn at the length of one half of the horse's third metacarpal bone. Boxplots show the mean (green triangle), median (black line in boxplot), and 1.5 times the Interquartile Range (whisker limits).

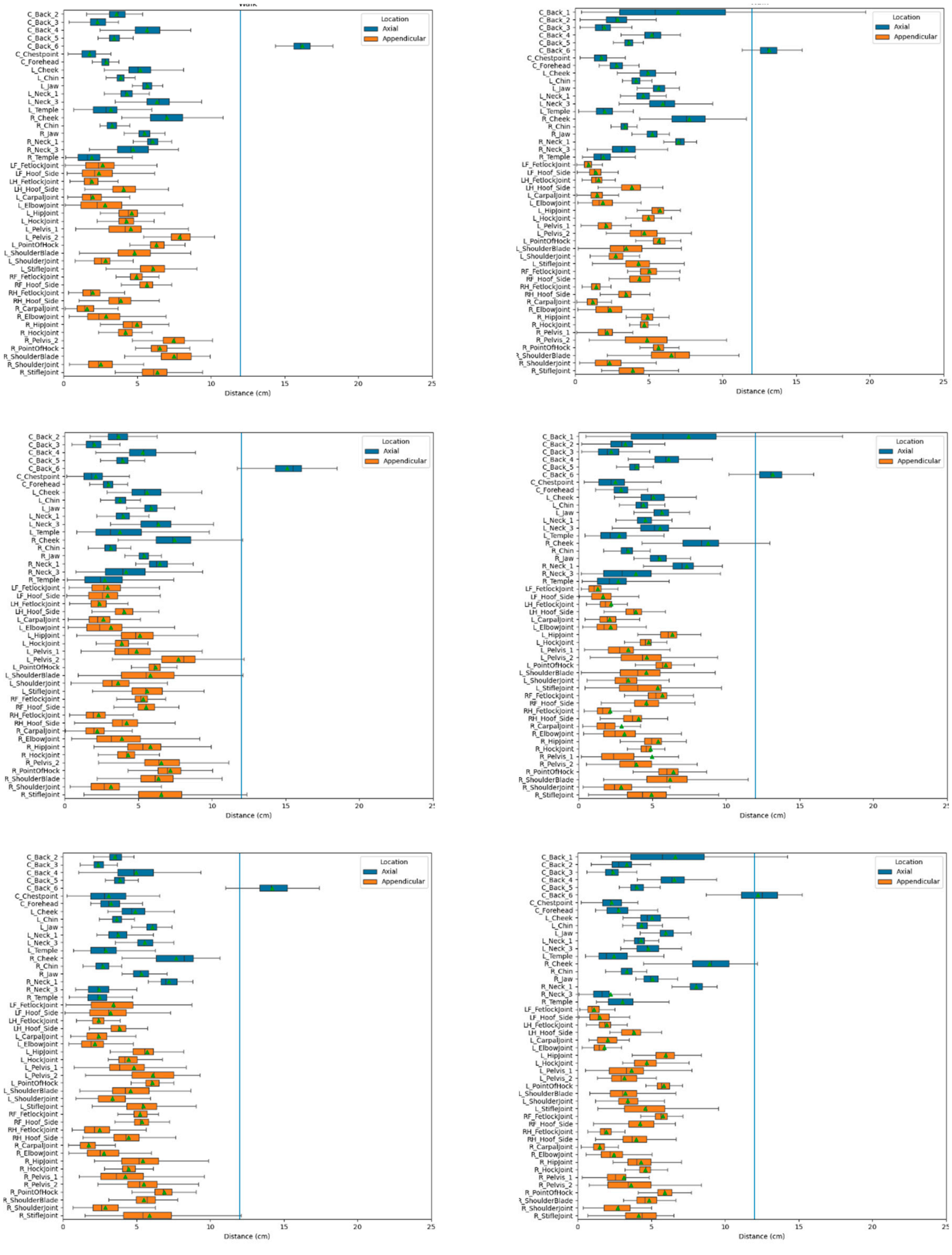

Figure S4: Inverse Kinematics Marker Error at the walk (top), trot (center), and canter (bottom) using predicted data for the networks trained on PFERD_base (Left) and on PFERD-SwRI_Horse (Right). A blue horizontal line is drawn at the length of one half of the horse's third metacarpal bone. Boxplots show the mean (green triangle), median (black line in boxplot), and 1.5 times the Interquartile Range (whisker limits).

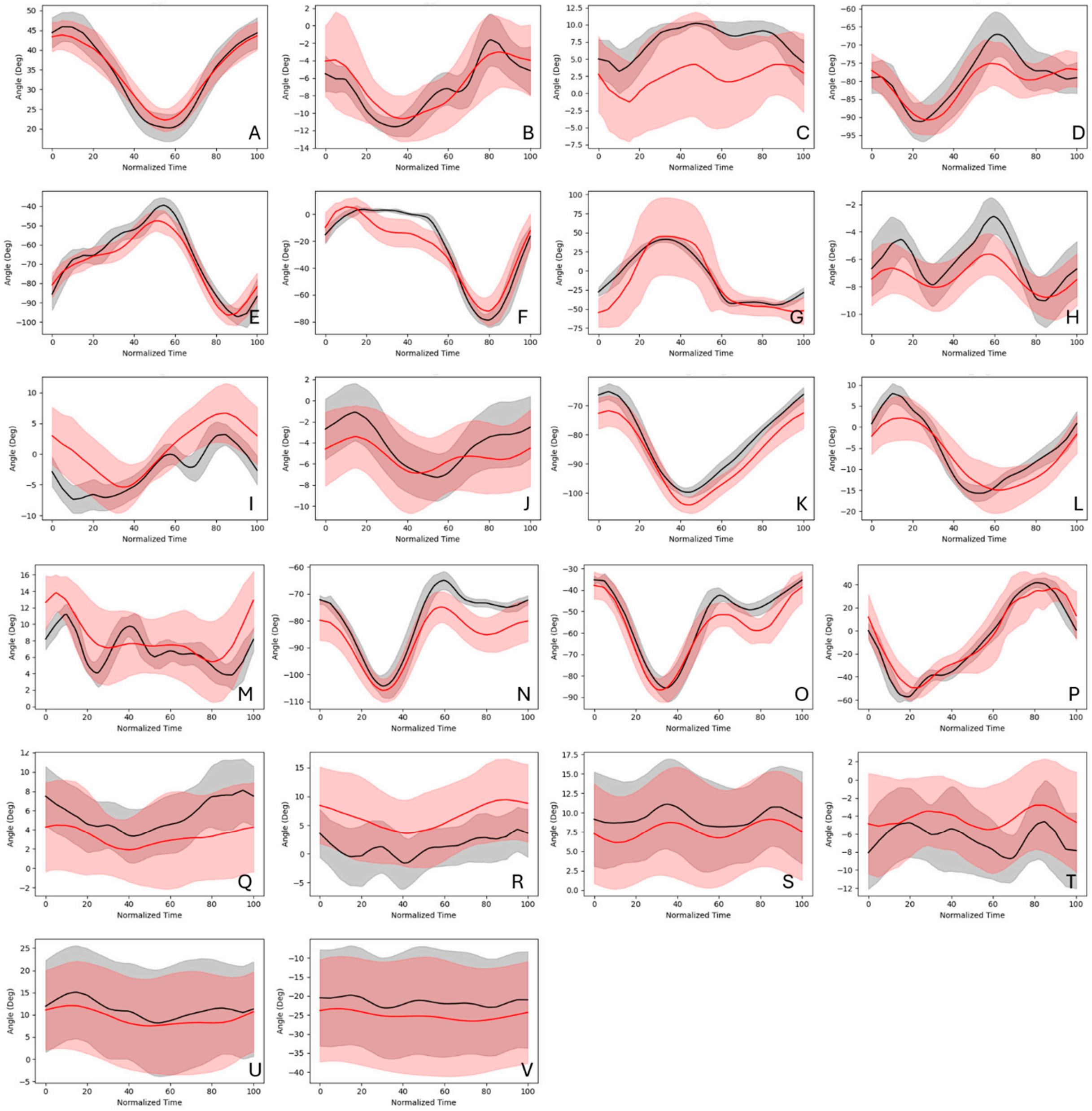

Figure S5. Examples of joint angles calculated using ground-truth marker locations (black) compared to those calculated using PFERD_base network predictions (red) across 24 strides of trot. The mean joint angle (solid line) and standard deviation (shaded) are shown for the following left side or axial skeleton joint angles: (A) Scapulothoracic (SCT) Z-direction, (B) SCT X-direction, (C) SCT Y-direction, (D) shoulder, (E) elbow, (F) carpus, (G) fore fetlock, (H) pelvis Z-direction, (I) pelvis X-direction, (J) pelvis Y-direction, (K) hip flexion (Z-direction), (L) hip X-direction, (M) hip Y-direction, (N) stifle, (O) hock, (P) hind fetlock, (Q) atlanto-occipital X-direction, (R) atlanto-occipital Y-direction. (S) atlanto-occipital Z-direction, (T) neck base X-direction, (U) neck back Y-direction, (V) neck base Z-direction.
